# Possible reversibility between epithelioid and sarcomatoid types of mesothelioma is independent of ERC/mesothelin expression

**DOI:** 10.1186/s12931-020-01449-2

**Published:** 2020-07-16

**Authors:** Masataka Kojima, Kazunori Kajino, Shuji Momose, Nadila Wali, May Thinzar Hlaing, Bo Han, Liang Yue, Masaaki Abe, Tomoaki Fujii, Katsuhisa Ikeda, Okio Hino

**Affiliations:** 1 Department of Pathology and Oncology, Juntendo University Faculty of Medicine, 2-1-1, Hongo, Bunkyo-ku, Tokyo, 113-8421 Japan; 2 Department of Otorhinolaryngology, Juntendo University Faculty of Medicine, 2-1-1, Hongo, Bunkyo-ku, Tokyo, 113-8421 Japan; 3 Department of Pathology, Saitama Medical Center, Saitama Medical University, 1981, Kamoda, Kawagoe, Saitama, 350-8550 Japan; 4 Division of Animal Genetics, Laboratory Animal Research Center, Institute of Medical Science, University of Tokyo, Tokyo, 108-8639 Japan

**Keywords:** Mesothelioma, ERC/mesothelin, Epithelioid type, Sarcomatoid type, Histological reversibility

## Abstract

**Background:**

Mesothelioma is histologically divided into three subgroups: epithelioid, sarcomatoid, and biphasic types. The epithelioid or sarcomatoid type is morphologically defined by polygonal or spindle-like forms of cells, respectively. The biphasic type consists of both components. It is not yet understood how histological differentiation of mesothelioma is regulated. ERC/mesothelin is expressed in most cases of the epithelioid type, but not in the sarcomatoid type of mesothelioma. Consequently, its expression is well correlated to the histological subtype. We hypothesized that ERC/mesothelin expression influences the histological differentiation of mesothelioma, and tested this hypothesis.

**Methods:**

We performed studies using the overexpression or knockdown of ERC/mesothelin in mesothelioma cells to examine its effect on cellular morphology, growth kinetics, or migration/invasion activity, in vitro. We then transplanted ERC/mesothelin-overexpressing and control cells into the intraperitoneal space of mice. We examined the effect of ERC/mesothelin overexpression on mouse survival and tumor phenotype.

**Results:**

In vitro cell culture manipulations of ERC/mesothelin expression did not affect cellular morphology or proliferation, although its overexpression enhanced cellular adhesion and the migration/invasion activity of mesothelioma cells. The survival rate of mice following intraperitoneal transplantation of ERC/mesothelin-overexpressing mesothelioma cells was significantly lower than that of mice with control cells. The histological evaluation of the tumors, however, did not show any morphological difference between two groups, and our hypothesis was not validated. Unexpectedly, both groups (ERC/mesothelin-overexpressing and control) of mesothelioma cells that were morphologically monophasic and spindle-like in vitro differentiated into a biphasic type consisting of polygonal and spindle-like components in the transplanted tumor, irrespective of ERC/mesothelin expression.

**Conclusions:**

These results suggested that the histological transition of mesothelioma between epithelioid and sarcomatoid types may be reversible and regulated not by ERC/mesothelin, but by other unknown mechanisms.

## Background

Expressed in Renal Carcinoma (ERC) was first identified in a renal cell carcinoma of an Eker rat [1, 2], and is the homolog of human mesothelin (*MSLN*) [3] or megakaryocyte potentiating factor [4]. ERC/mesothelin is expressed in normal mesothelium, and its expression is enhanced in epithelioid mesothelioma, ovarian cancer, and other malignancies [3–9]. Functionally, it is reported to enhance cellular adhesive or invasive activities [10–12]. As for cellular proliferation, several groups have described how ERC/mesothelin has positive effects [13–15]; however, the other groups report no significant effects [11, 12].

Mesothelioma most commonly arises in the pleura, and, at much lower frequency, also occurs in the peritoneum, pericardium, and tunica vaginalis testis. It is histologically divided into three subgroups: epithelioid, sarcomatoid, and biphasic (containing two components) types, consisting of approximately 60, 20, and 20%, respectively, of the pleural mesothelioma [16–18]. Morphologically, mesothelioma cells in the epithelioid type take on a polygonal or cobblestone-like form, and those in the sarcomatoid take on a spindle-like shape. The median survival time after surgical therapy is 15–19, 4–10, and 10–12 months [17–19] in these groups, respectively, indicating that the prognosis of the sarcomatoid type is poorer than that of the epithelioid one. At present, the underlying mechanism that defines the histological differentiation into these subgroups is not yet known.

ERC/mesothelin is expressed in most (75–100%) cases of the epithelioid, but not sarcomatoid, type [20–22]. Consequently, ERC/mesothelin expression and the histological subtype of mesothelioma are well correlated. We hypothesized that the expression status of ERC/mesothelin influences the morphological phenotype of mesothelioma. To test this hypothesis, we examined the effects of ERC/mesothelin overexpression or knockdown on cell morphology, as well as growth kinetics, adhesion, and migration/invasion of mesothelial cells in vitro. We then intraperitoneally transplanted ERC/mesothelin-overexpressing and control cells into mice, and examined the effect of ERC/mesothelin on mouse survival and tumor phenotype.

## Methods

### Cell lines and antibodies

**Fig. 1:**
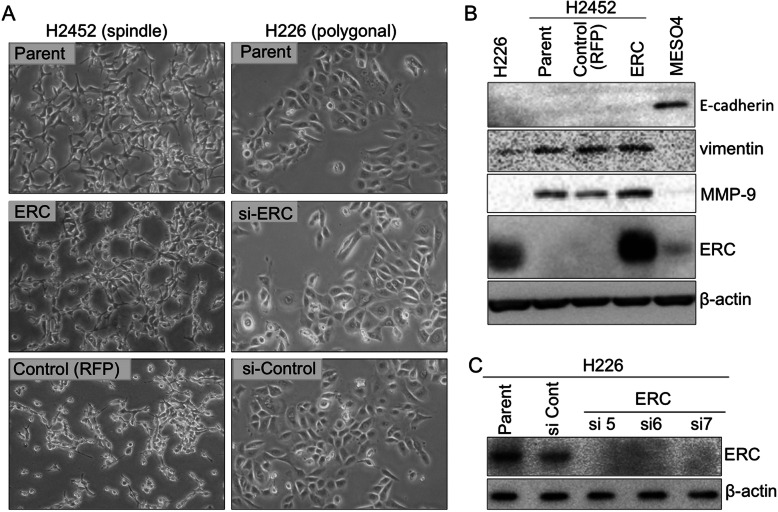
Effects of ERC/mesothelin expression on mesothelioma cells. **a**, Effects of ERC/mesothelin overexpression (left column) or knockdown (right column) on the morphology of H2452 or H226 cells, respectively, as observed by phase-contrast microscopy (objective lens × 10). Top panels: parental H2452 or H226; Middle panels: H2452 overexpressing ERC/mesothelin or H226 treated with siRNA of ERC/mesothelin; Bottom panels: H2452 or H226 treated with control vector or control siRNA. **b**, Effects of ERC/mesothelin overexpression on the expression of E-cadherin, vimentin and MMP-9 assessed by western blotting. Note that MESO4 (ACC-MESO-4) and H226 cells were used as positive and negative controls, respectively, for E-cadherin. **c**, Effects of three siRNAs of ERC/mesothelin (si5, si6, si7) on expression. RFP, red fluorescent protein; MMP-9, matrix metalloproteinase-9

The human mesothelioma cell lines NCI-H2452 (H2452) and NCI-H226 (H226) were obtained from the American Type Culture Collection (ATCC; Rockville, MD, USA), and ACC-MESO-4 (MESO4), which was established at the Aichi Cancer Research Center Institute [23], was obtained from RIKEN BioResource Center (RIKEN BRC; Tokyo, Japan). H226 and MESO4 expressed endogenous ERC/mesothelin, but H2452 did not (Fig. 1b). All cell lines were cultured in RPMI-1640 medium supplemented with 10% fetal calf serum (FCS) at 37 °C in a 95% air/5% CO_2_ atmosphere.

A mouse monoclonal anti-human C-ERC/mesothelin antibody, 22A31, has been described previously [24]. Other antibodies used in this study included mouse monoclonal anti-vimentin (clone V9), anti-cytokeratin (clone AE1/AE3), anti–E-cadherin (clone NCH-38), and anti–Ki-67 (clone MIB-1; Dako, Glostrup, Denmark); rabbit polyclonal anti-integrin α5 (#4705), anti-integrin β1 (#4706), rabbit monoclonal anti-matrix metalloproteinase-9 (MMP-9) (clone D603H; Cell Signaling Technology Japan, Tokyo, Japan); rabbit polyclonal anti-Twist (H81) (Santa Cruz Biotechnology, CA, USA); and rabbit polyclonal anti-ZEB1 (HPA027524; Sigma–Aldrich/ Merck, KGaA, Darmstadt, Germany).

### Western blotting

A cellular lysate (30 μg) was harvested in 2% SDS, 10% glycerol, 50 mM Tris-HCl (pH 6.8), and 100 mM dithiothreitol. After boiling for 2 min, samples were electrophoresed on 10% polyacrylamide gels and transferred onto polyvinylidene difluoride membranes (Immobilon-P, Merck Millipore, Burlington, MA, USA). The membranes were blocked in 1% skim milk in phosphate-buffered saline (PBS) with 0.1% Tween-20 (PBS-T) for 1 h at room temperature. The membranes were then incubated with anti–C-ERC/mesothelin (1 μg/mL), anti-vimentin (1:200), anti–E-cadherin (1:250), anti-integrin α5, anti-integrin β1, anti-MMP-9 (1:1000), anti-Twist (1:200), or anti-ZEB1 (1:250) at room temperature for 1 h in 1% skim milk in PBS-T. Goat anti-mouse or anti-rabbit Ig conjugated with peroxidase labeled-dextran polymer (Envision+ System, Dako) was used as a secondary antibody at a dilution of 1:100 in 1% skim milk in PBS-T at room temperature for 1 h. An ECL detection system (GE Healthcare, Chicago, IL, USA) was employed to visualize proteins on membranes. ECL signals were detected and quantified by a ChemiDoc MP imaging analyzer (Bio-Rad, Tokyo, Japan). The expression level of ß-actin was used as an internal control for the determination of equal loading.

### Immunohistochemistry

Three–micrometer thick tissue sections were prepared from formalin-fixed, paraffin-embedded specimens. After deparaffinization, tissue sections were heated in 10 mM citrate buffer (pH 6) for antigen retrieval and treated with 3% hydrogen peroxide. The sections were incubated with primary antibodies diluted in Tris-buffered saline with 0.1% Tween 20 overnight at 4 °C. Anti–E-cadherin, anti–Ki-67, and anti-vimentin antibodies were diluted at 1:200, anti-AE1/AE3 antibodies was diluted at 1:400, and anti-Twist and anti-ZEB1 were diluted at 1:100. Anti–C-ERC/mesothelin antibody was used at 2 μg/mL. Immunohistochemistry (IHC) using mouse monoclonal antibodies was performed with a Histofine Mouse Stain kit (Nichirei, Tokyo, Japan), and that using the anti-rabbit antibody was performed with an Envision+ System secondary antibody (Dako). Diaminobenzidine was used as the substrate.

### ERC/mesothelin overexpression in H2452 cells using lentivirus vector

HEK293T, which was used as the packaging cell line, was cotransfected with Precision LentiORF for *MSLN* and trans-lentiviral packaging vectors (Thermo Scientific Open Biosystems, Waltham, MA, USA). A LentiORF-MSLN vector encoded ERC/mesothelin and Turbo green fluorescent protein (GFP). A vector in which ERC/mesothelin was replaced with Turbo red fluorescent protein (RFP) was used as a negative control. Sixteen hours after transfection, we microscopically confirmed the presence of GFP- or RFP-positive cells, and the medium was changed to that with 5% FCS. Forty-eight hours after medium change, supernatants were harvested and their infectivity on H2452 cells was titrated by counting the number of TurboGFP- or TurboRFP-positive cells. To establish stable ERC/mesothelin- or RFP-expressing cells, we infected H2452 cells with the titrated supernatant at a multiplicity of infection of 2.0, and selected cells that were resistant to 2.0 μg/mL blasticidin S.

### SiRNA transfection to knock down ERC/mesothelin in H226 cells

ON-TARGET plus Human *MSLN* siRNAs, including (5′-CAUUGGACCUGCUGCUAUU-3′), (5′-ACAUGAACGGGUCCGAAUA-3′), and (5′-GAUGAGCUCUACCCACAAG-3′), and ON-TARGET plus Non-targeting Pool siRNA (negative control) were purchased from Dharmacon/GE Healthcare (Lafayette, CO, USA). H226 cells were seeded at 7.5 × 10^4^ in 3-cm plates. Twenty-four hours later, the cells were transfected with 10 nM siRNA or with transfection reagent (Lipofectamine RNAiMAX; Invitrogen, Carlsbad, CA, USA) alone. In the following 96 h, cellular morphology and proliferative states were observed. For western blotting, cell lysates were harvested 48 h after siRNA transfection.

### Cell adhesion assay

Flat 96-well plates were coated with Matrigel (Corning, Corning, NY, USA; 100 μg/mL, 100 μL/well) or fibronectin (Corning; 20 μg/mL, 100 μL/well) and then incubated at 37 °C in a 5% CO_2_ atmosphere for 1 h. The coated wells were washed twice with 0.1% bovine serum albumin (BSA), blocked with 0.5% BSA for 1 h at 37 °C in a 5% CO_2_ atmosphere, and then washed with 0.1% BSA again. Cells were seeded at 2 × 10^4^ cells/well and incubated for 1 h at 37 °C in a 5% CO_2_ atmosphere. They were then washed twice with PBS, fixed with 4% paraformaldehyde for 10 min, and then washed with PBS again. The cells were stained with 1% Crystal Violet at room temperature for 10 min. Solubilization of Crystal Violet was performed in 33% acetic acid, and the absorbance was measured at 550 nm. The measurements were conducted in triplicate for each experimental group.

### Scratch wound migration/invasion assays

IncuCyte ImageLock 96-well plates (Essen BioScience, Tokyo, Japan) were coated with Matrigel at 100 μg/mL and incubated overnight at 37 °C. Cells were seeded at 6 × 10^4^ cells/well and allowed to adhere on top of a thin layer of Matrigel for 4 h at 37 °C. A wound was created with a 96-well WoundMaker (Essen Bioscience). More Matrigel (6 mg/mL, 50 μL/well) was overlaid on top of the cells to create a three-dimensional matrix. Finally, an IncuCyte ZOOM live-cell imaging and analysis platform (Essen Bioscience) was used to quantify invading cells in the wound area.

### Cell proliferation assay

Cells (1 × 10^3^ cells/well) in RPMI-1640 with 10% FCS were seeded in flat 96-well dishes, and incubated at 37 °C in a 5% CO_2_ atmosphere. The area of proliferating cells was scanned and quantified by the IncuCyte ZOOM system (Essen Bioscience) every 3 h for 96 h.

### Animal experiments

All in vivo studies were approved by the Institute Animal Care and Use Committee of Juntendo University. Female BALB/c athymic nude (BALB/c nu/nu) mice at 6 weeks of age were purchased from Charles River Japan (Yokohama, Japan). After 14 days of acclimatization, 2.5 × 10^6^ of ERC/mesothelin-overexpressing or control H2452 cells were injected into the intraperitoneal (IP) space of the mice. The mice were euthanized when they showed moribund sign, or on day 70 after injection. The IP space was opened, and any tumors present were harvested. All mice were maintained under specific pathogen-free conditions.

### Statistical analysis

We used Student’s *t* test to evaluate differences between two groups. Data represent the mean ± standard deviation (SD). The survival rate of mice was compared by the Kaplan–Meier method, and log-rank tests were used to estimate statistical significance between two groups. *P* < 0.05 was considered statistically significant.

## Results

### The effects of ERC/mesothelin expression on mesothelioma cells in vitro

**Fig. 2:**
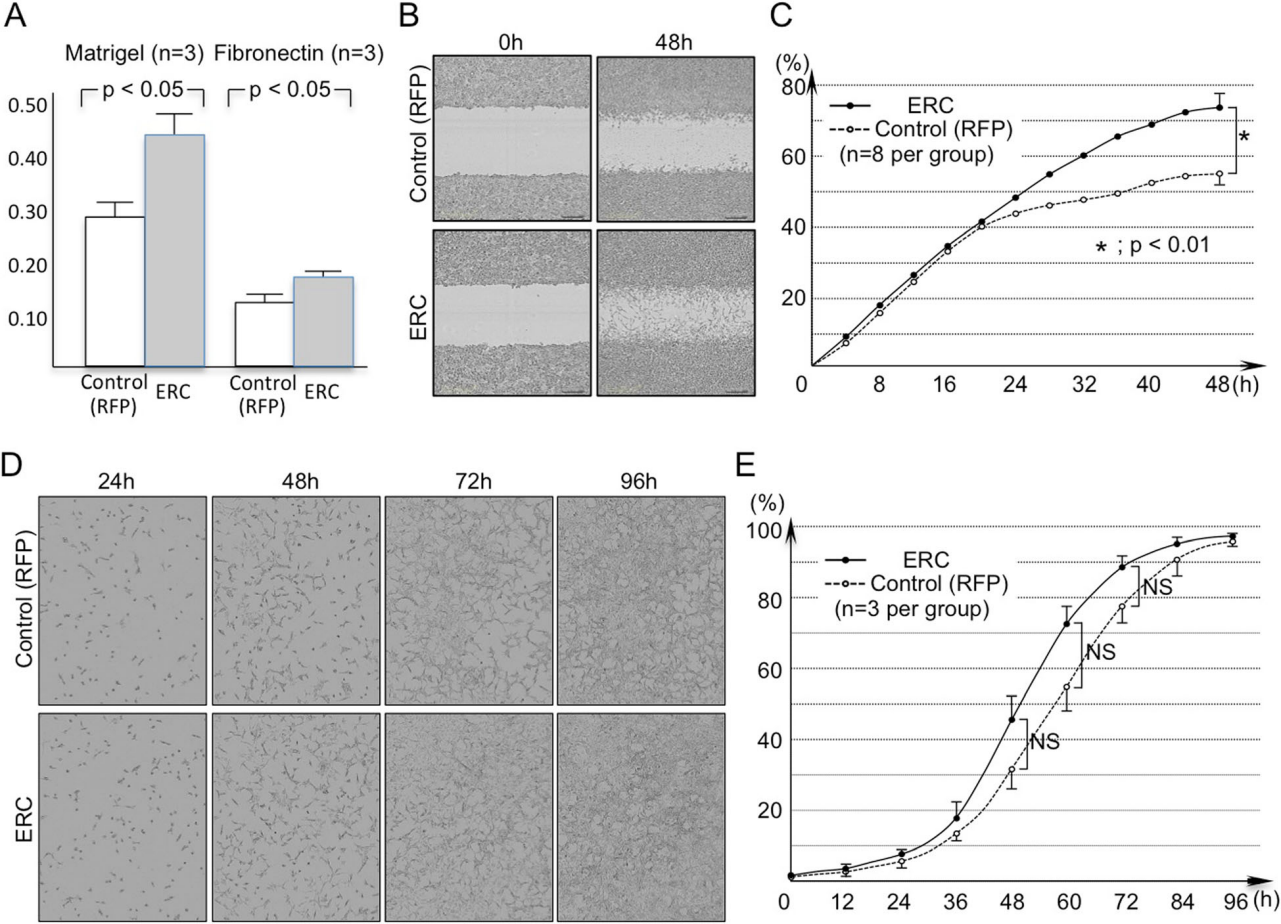
Effects of ERC/mesothelin overexpression on cell adhesion to extracellular matrix (**a**), invasion/migration (scratch assay) (**b**, **c**), and cell proliferation (**d**, **e**) of H2452 cells treated with ERC/mesothelin-overexpressing or control vectors. **b** and **d**, cells observed under a phase-contrast microscope. **c** and **e**, quantification of invading/migrating (**c**) and proliferating (**e**) cells. Solid line, ERC/mesothelin (+) cells; dotted line, control cells. Numbers (%) in the vertical axis indicate the ratio of the area occupied by cells relative to the whole area of interest. In E, there was no significant difference between the two groups for all time points to 96 h. n.s. not significant; RFP, red fluorescent protein

We hypothesized that the expression of ERC/mesothelin influences the morphology of cells, as its expression is well correlated to the histological subtypes of mesothelioma. Thus, we examined the effect of ERC/mesothelin overexpression or knockdown on cellular morphology. As shown in Fig. 1a, ERC/mesothelin overexpression in H2452 (spindle-shaped) or knockdown in H226 (polygonal) cells did not affect cell morphology. The overexpression or knockdown of ERC/mesothelin was confirmed in Fig. 1b and c. The manipulation of ERC/mesothelin expression did not have any effect on epithelial–mesenchymal transition (EMT) markers such as E-cadherin, vimentin (Fig. 1b), or ZEB1, or Twist (Additional file 1: Figure S1). We then examined the effects of ERC/mesothelin on cellular activities. We found that ERC/mesothelin overexpression enhanced cellular adhesion (Fig. 2a) and migration/invasion (Fig. 2b and c) with regard to the extracellular matrix (ECM), but did not influence cellular proliferation (Fig. 2d and e). The expression of MMP-9 was enhanced in ERC/mesothelin-overexpressing cells (Fig. 1b), but that of integrin α5 and integrin β1 remained unchanged (Additional file 1: Figure S1).

### The effect of ERC/mesothelin expression on mesothelioma cells in vivo

**Fig. 3:**
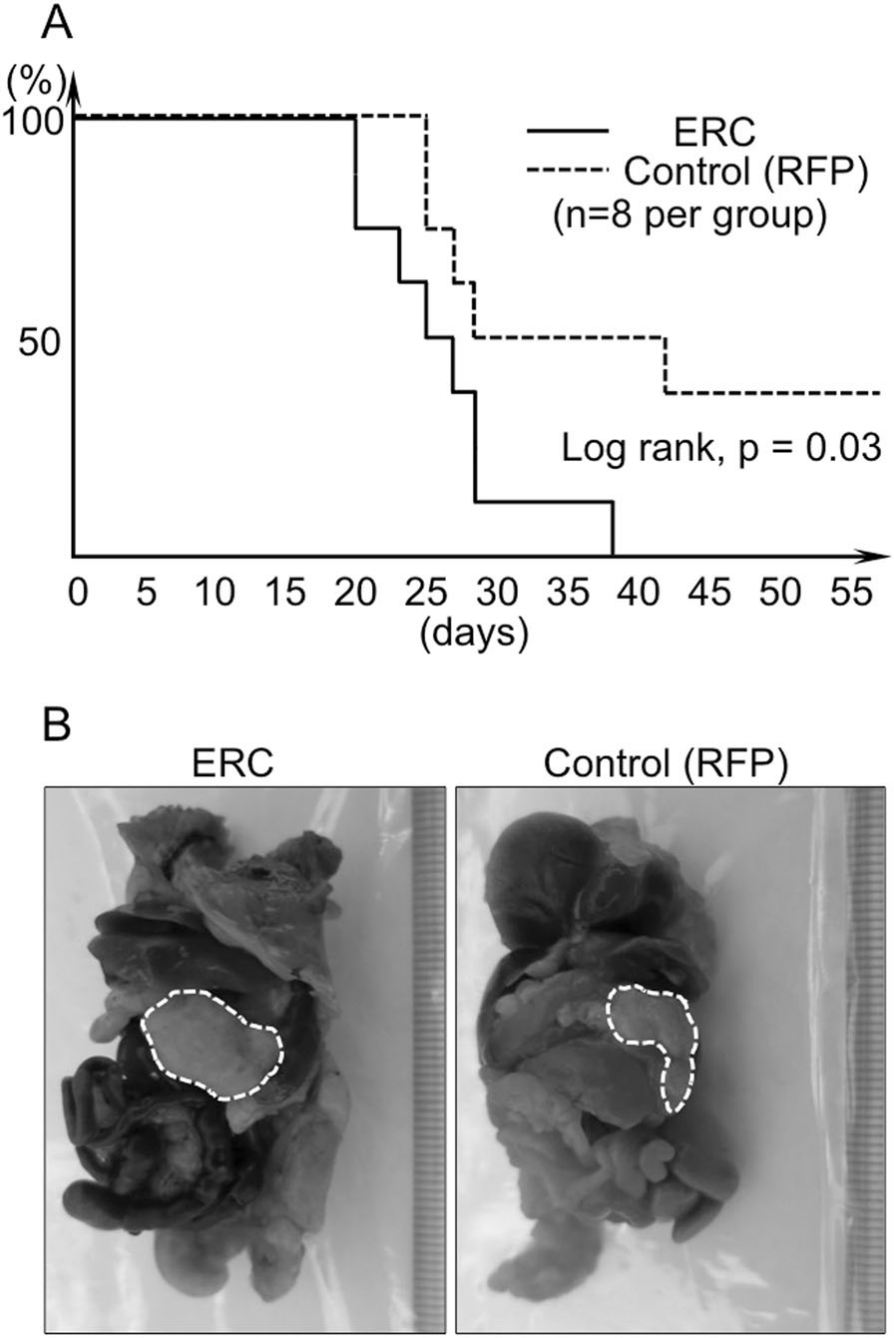
Tumor development in mice transplanted intraperitoneally with ERC-overexpressing or control H2452 cells. **a**, Survival rate of mice transplanted with ERC-overexpressing (solid line) or control (dashed line) H2452 cells. **b**, Representative macroscopic findings of ERC/mesothelin (+) or control tumors. Tumors are encircled by white dashed lines

Following transplantation of ERC/mesothelin-overexpressing H2452 cells into the IP space of nude mice (*n* = 8), we observed that the overall survival period of these mice was significantly shorter than those transplanted with control H2452 (n = 8; Fig. 3a). By 40 days’ post-transplantation, all eight mice transplanted with ERC/mesothelin-overexpressing H2452 were euthanized because of moribund sign, or found dead without the sign. On the other hand, three out of eight control mice survived until 70 days’ post-transplantation, when they were euthanized and tumors were not found. All 13 mice that were euthanized or found dead before 70 days had developed tumors in IP spaces. Thus, the difference in survival rate was caused by a difference in transplantation efficiency; 100% (8/8) vs. 62.5% (5/8) in ERC/mesothelin-overexpressing vs. control cells, respectively. Representative tumors from the two groups are shown in Fig. 3b; the length of tumors in the two groups did not show any significant difference (eight ERC/mesothelin-overexpressing tumors, 13.6 ± 2.9 mm; five controls 12.2 ± 4.4 mm).

### Histological differentiation of mesothelioma is independent of ERC/mesothelin expression

**Fig. 4:**
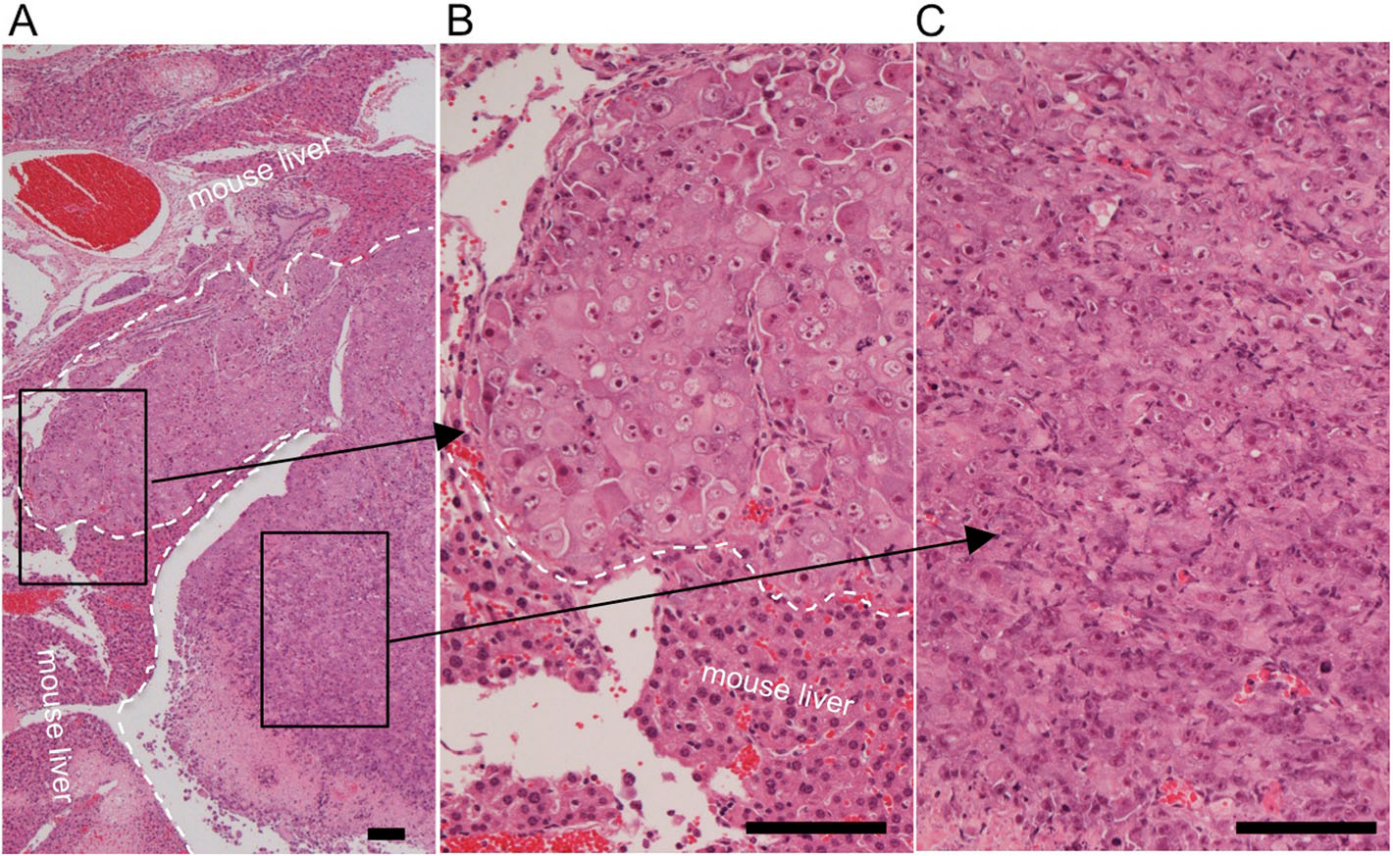
Histology of a representative tumor derived from transplanted H2452 cells that overexpressed ERC/mesothelin. **a**, Hematoxylin & eosin (HE)-stained findings at lower magnification. Higher magnification images of the boxed areas are shown in **b** and **c**. The white dashed lines demarcate the border between invading mesothelioma cells and mouse liver. Scale bars, 100 μm in all three figures

**Fig. 5:**
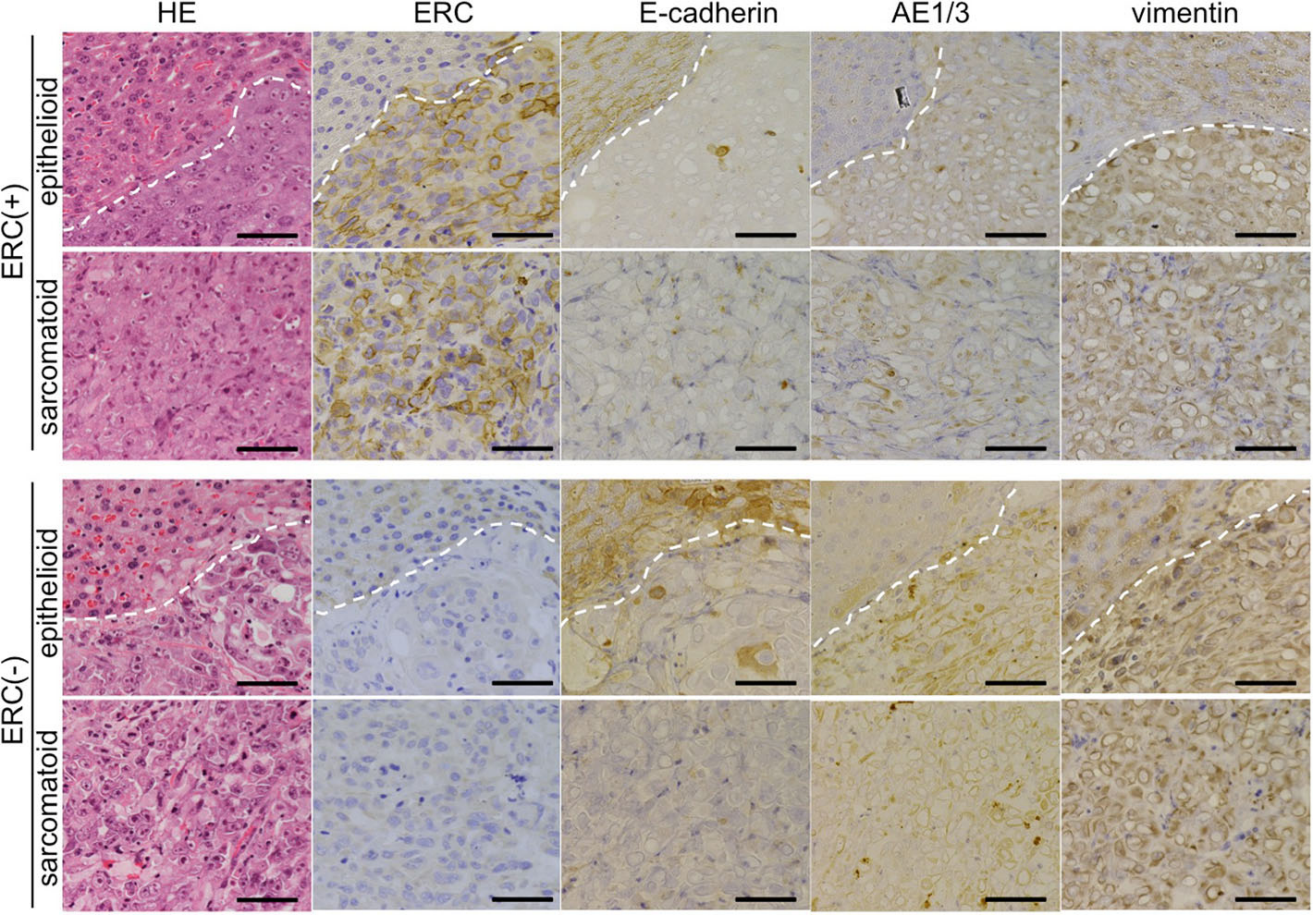
Hematoxylin & eosin (HE) staining and immunostaining for ERC/mesothelin, E-cadherin, AE1/AE3 and vimentin, in epithelioid or sarcomatoid areas in both ERC/mesothelin-overexpressing and control tumors derived from H2452. In images of epithelioid areas (top and third rows), the white dashed lines demarcate the border between invading mesothelioma cells (lower right) and mouse liver (upper left). Scale bars, 50 μm in all figures

**Fig. 6:**
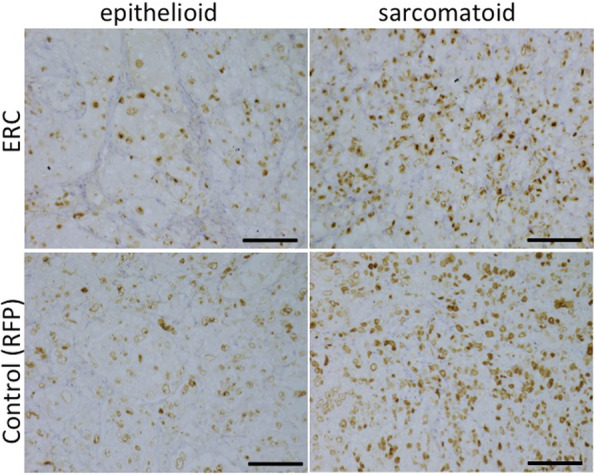
Immunostaining for Ki-67 in epithelioid or sarcomatoid areas in both ERC/mesothelin-overexpressing and control tumors derived from H2452 cells. Representative areas in each tumor are shown. Scale bars, 100 μm in all figures

In transplantation experiments, we did not find any histological differences between tumors derived from ERC/mesothelin-overexpressing H2452 or control cells. Unexpectedly, we detected biphasic mesothelioma in ERC/mesothelin-overexpressing tumors (Fig. 4) as well as in the control. In Fig. 4b, cells invading into the mouse liver tissue demonstrated a polygonal pattern, whereas in Fig. 4c, the cells exhibited a spindle-shape. The same pattern was observed in the control group. In both cases, a polygonal pattern was observed only at the invading front of the tumor. Figure 5 and Figure S2 (Additional file 1) showed that, irrespective of ERC/mesothelin expression and histological subtypes, the E-cadherin stain was negative, while those for AE1/AE3, vimentin, ZEB1 and Twist were weakly positive in tumors derived from H2452. There was no difference in the IHC staining pattern of EMT markers between ERC/mesothelin positive and negative tumors. The proliferative activity of mesothelioma cells, evaluated by number of Ki-67 positive cells, was also not influenced by ERC/mesothelin expression (Fig. 6). Ki-67 positivity was 20–30% or 60–80% in epithelioid and sarcomatoid areas, respectively, both in ERC/mesothelin-overexpressing and control groups.

## Discussion

We initially hypothesized that ERC/mesothelin influences the morphology of mesothelioma, because its expression correlated well with morphological subtypes of mesothelioma. Our hypothesis was shown to be invalid by the negative experimental data. We showed this negative data because we considered it worthwhile to be published. To date, no report exists that discusses the functional relationship between the ERC/mesothelin expression and histological differentiation in mesothelioma. The expression of EMT markers, such as E-cadherin, vimentin (Figs. 1b and 5), ZEB1, or Twist (Additional file 1: Figure S1), was also not influenced by ERC/mesothelin overexpression or knockdown. ERC/mesothelin overexpression, however, enhanced the cell adhesion and migration/invasion activity of cells with regard to the ECM in vitro (Fig. 2a-c). To explore the reason for such effects, we evaluated expression levels of MMP-9 that is associated with enhanced migration/invasion of ERC/mesothelin-expressing cells [12]. Figure 1b shows that the expression of MMP-9 was enhanced in ERC/mesothelin-expressing cells, and it possibly caused the enhanced migration/invasion activity of the ERC/mesothelin-expressing cells in our study. We tried to detect MMP-9-positive cells in transplanted tumors by IHC, but we could not detect specific signals in both of ERC/mesothelin-expressing and control cells (data not shown). The expression of cell adhesion molecules such as integrin α5 or integrin β1 remained unchanged (Additional file 1: Figure S1).

Our previous experiments showed that the transplantation efficiency of the unmanipulated, parental H2452 cells (2.5 × 10^6^) was not 100%, but 60–70% in 70 days (data not shown). In this study, control cells expressing RFP formed tumors in 62.5% (5/8) of mice, and a frequency was almost identical to that of unmanipulated cells. ERC/mesothelin-expressing cells, on the contrary, formed tumors in 100% of mice, and such reinforced transplantation efficiency in vivo was probably caused by the enhanced adhesion and migration/invasion activities observed in vitro. Proliferation activities did not differ between the two groups, both in vitro and in vivo (Fig. 2d, e, and 6).

According to the World Health Organization classification [25], the most commonly encountered patterns of epithelioid mesothelioma are further divided into solid, tubulopapillary, and trabecular subtypes. The epithelioid component shown in Fig. 4b took the form of a solid pattern. To rule out that the biphasic phenotype was caused by the oligo-clonality of H2452 cells, we performed single-cell cloning of unmanipulated H2452 cells, and transplanted the cloned cells into mice. We obtained similar reproducible results demonstrating biphasic tumors with both polygonal and spindle-shape patterns (data not shown).

**Fig. 7:**
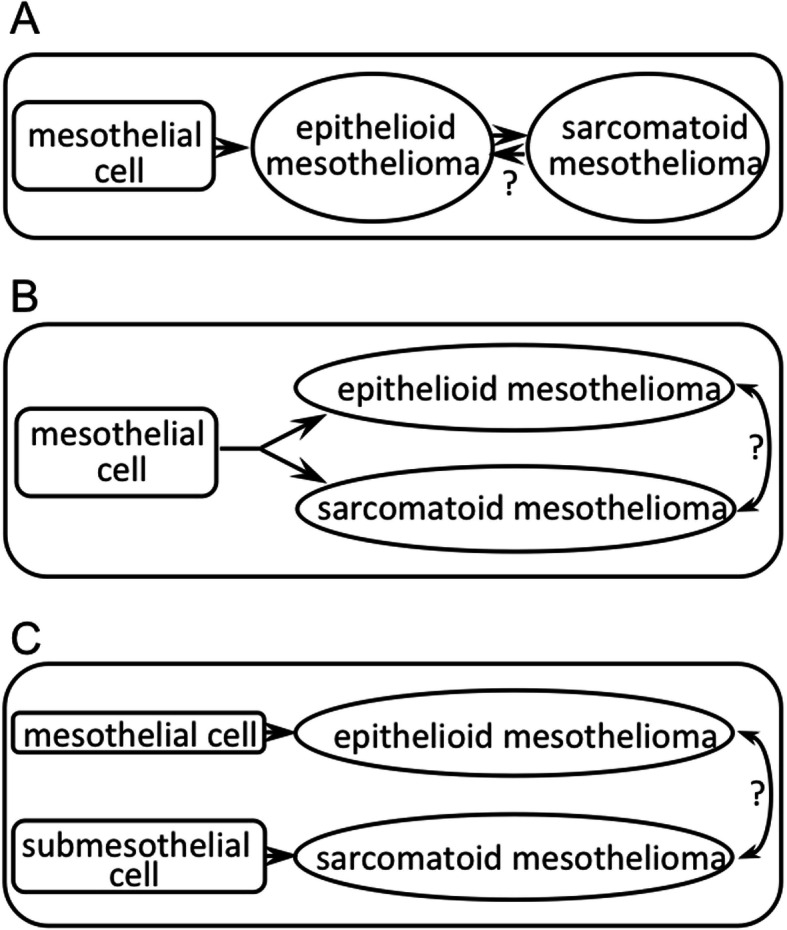
Three possible pathways (**a**, **b**, **c**) for progression from precursor cells to epithelioid or sarcomatoid mesothelioma. Question marks (?) in **a**, **b**, and **c** indicate possible reversibility of the two subtypes

Mesothelioma is reported to originate from normal mesothelial cells [26] or submesothelial, mesenchymal cells [27, 28]. At present, it is generally considered that the sarcomatoid type progresses from the epithelioid type as shown in Fig. 7a. However, it is still possible that both occur from the same precursor cell via independent pathways (Fig. 7b), or that the two types are derived from different precursor cells (Fig. 7c). Our study showed that H2452 demonstrating a monophasic spindle-like shape in vitro developed into biphasic mesothelioma with both epithelioid (polygonal) and sarcomatoid (spindle) components in transplanted mice. This suggests that the transition between the epithelioid and sarcomatoid types may be reversible, as shown by “?” in Fig. 7a-c.

In malignancies derived from epithelial organs, such as lung or kidney carcinomas, a sarcomatous change is considered to occur as the consequence of a progression to more malignant stages [29, 30]; with further progression, epithelial components are replaced by sarcomatous ones. The term “carcinosarcoma” or “sarcomatoid carcinoma” refers to biphasic states in which both the epithelial and sarcomatous components coexist. The frequency of carcinosarcoma is very low, less than 1% of all lung or kidney malignancies [31, 32]. In contrast, in cases of mesothelioma, the biphasic type represents as much as 20% of all cases [16–18]. This high frequency of the biphasic type suggests that epithelioid and sarcomatoid types may be interchangeable or reversible.

H2452 (NCI-H2452) is a cell line established from epithelioid mesothelioma according to the ATCC. In in vitro culture, cells exhibited a fibroblastic form and behaved like sarcomatoid cells. This phenomenon also implied the histological reversibility of mesothelioma. H2452 has multiple mutations in tumor suppressor genes, including a missense mutation in *BAP1* [33], a truncation of *p53* [34], and a homozygous deletion of *CDKN2A* [35] and *NF2* [36]. It was significant for us that the cell line, which harbored mutations and showed monophasic morphology in cell culture, became biphasic in vivo.

Several reports have investigated the expression of EMT and mesenchymal–epithelial transition (MET) markers in epithelioid and sarcomatoid mesotheliomas [37, 38]. The significance of EMT/MET in the development of mesothelioma is still controversial. In our study, the histological differentiation of H2452 to polygonal and spindle-shaped components occurred without changes in expression of EMT markers such as E-cadherin, AE1/AE3, vimentin (Fig. 5), and ZEB1 and Twist (Additional file 1: Figure S2).

Our data suggested that the morphological differentiation of mesothelioma is reversible. What kind of mechanisms are regulating it? We must consider not only intrinsic factors of mesothelioma cells, but also microenvironmental factors associating with them. As for the intrinsic ones, multiple studies showed that normal mesothelial cells have the ability to change phenotype and behave like multipotent stem cells that can differentiate to smooth muscle cells or fibroblasts [39–41]. Considering these findings, it is possible that mesothelioma maintains the characteristics of multipotency even after the acquirement of a malignant character. In human mesothelioma cases, CAM5.2 and AE1/AE3, both of which are usually used as the epithelial markers, are expressed in the sarcomatoid type [42, 43], and vimentin, which is one of the mesenchymal markers, is expressed in the epithelioid one [21]. Therefore, the expression of molecules conventionally used as epithelial or mesenchymal markers are not well associated with the morphology of mesothelioma. There should be some other unknown molecules that regulate its differentiation. Several studies analyzed differences in gene expression patterns between epithelioid and sarcomatoid subtypes. Lopez-Rios et al. reported that uroplakins 1B, 3B and kallikrein 11 are more prominently expressed in the epithelioid types [44]. De Rienzo et al. showed that molecules associated with tyrosine kinase signaling, germ cell development, and regulation of cell proliferation are upregulated in the epithelioid mesothelioma [45]. At present, it is not known whether any of these molecules are working as the regulating factors for the differentiation of mesothelioma. They compared gene expression in mesotheliomas with different genetic backgrounds, that could induce some nonspecific effects. We are currently examining the expression and mutation patterns of genes in epithelioid or sarcomatoid components with an identical genetic background, using our experimental systems and laser microdissection. As for the microenvironmental factors relevant to the differentiation of mesothelioma, Fig. 4 showed interesting findings. The transplanted H2452 took a polygonal, epithelioid pattern at the invasion front where it contacted with the host hepatocytes, and in the distant area from the front the cell took a spindle-like, sarcomatoid pattern. Polygonal host hepatocytes seemed to have some effect on the morphology of the adjacent mesothelioma cells with unknown mechanisms. Matsukuma et al. observed that metastatic cancer to the pancreas showed the morphology resembling to that of primary pancreatic cancer, and proposed the concept of “mimicry” of the metastatic cells to the primary carcinoma in the site of metastasis [46]. Shepherd and Hall also reported the similar findings in metastatic cancer in the colon [47]. The findings in Fig. 4 may be reflecting the phenomenon of “mimicry”, although its molecular mechanisms are not yet known.

## Conclusions

Our initial hypothesis that ERC/mesothelin regulates the histological differentiation of mesothelioma was not supported by the experimental data. Instead, mesothelioma cells with a monophasic morphology in culture developed into biphasic cells in a mouse model, regardless of the expression of ERC/mesothelin. These results suggested that the histological differentiation of mesothelioma (epithelioid vs. sarcomatoid) may be reversible and regulated by mechanisms other than those for ERC/mesothelin or EMT/MET. Further molecular studies both of intrinsic factors in mesothelioma cells and microenvironmental factors associating with them are required to elucidate the the mechanisms of differentiation of mesothelioma.

## Supplementary information

**Additional file 1 **: **Figure S1.** Effects of ERC/mesothelin overexpression on the expression of EMT markers (ZEB1 and Twist), Integrins α5 and β1, assessed by western blotting. **Figure S2.** HE staining and immunostaining for ZEB1 and Twist, in the epithelioid or sarcomatoid areas in both of ERC/mesothelin-overexpressing and control tumors derived from H2452. In figures of epithelioid area (top and third figures in each column), the white dotted lines demarcate the border between the invading mesothelioma cell (lower) and mouse liver (upper). Scale bars, 50 μm in all figures.

## Data Availability

Data sharing is not applicable to this article as no datasets were generated or analysed during the current study.

## Abbreviations

ATCC: American type culture collection
BAP1: BRCA1 associated protein-1
CDKN2A: Cyclin-dependent kinase inhibitor 2A
ECM: Extracellular matrix
EMT: Epithelial-mesenchymal transition
ERC: Expressed in renal carcinoma
FCS: Fetal calf serum
GFP: Green fluorescent protein
IHC: Immunohistochemistry
MET: Mesenchymal-epithelial transition
MMP-9: Matrix metalloproteinase-9
MSLN: Mesothelin
NF2: Neurofibromin 2
PBS: Phosphate-buffered saline
PBS-T: PBS with 0.1% Tween-20
RFP: Red fluorescent protein
RIKEN BRC: RIKEN bio-resource center
SDS: Sodium dodecyl sulfate
SD: Standard deviation
siRNA: Small interfering RNA
ZEB1: Zinc finger E-box-binding homeobox 1
