# Supplementary figures and images for: Possible reversibility between epithelioid and sarcomatoid types of mesothelioma is independent of ERC/mesothelin expression
Source: Respir Res. 2020 Jul 16;21:187. doi: 10.1186/s12931-020-01449-2 (PMC7364551; doi:10.1186/s12931-020-01449-2)

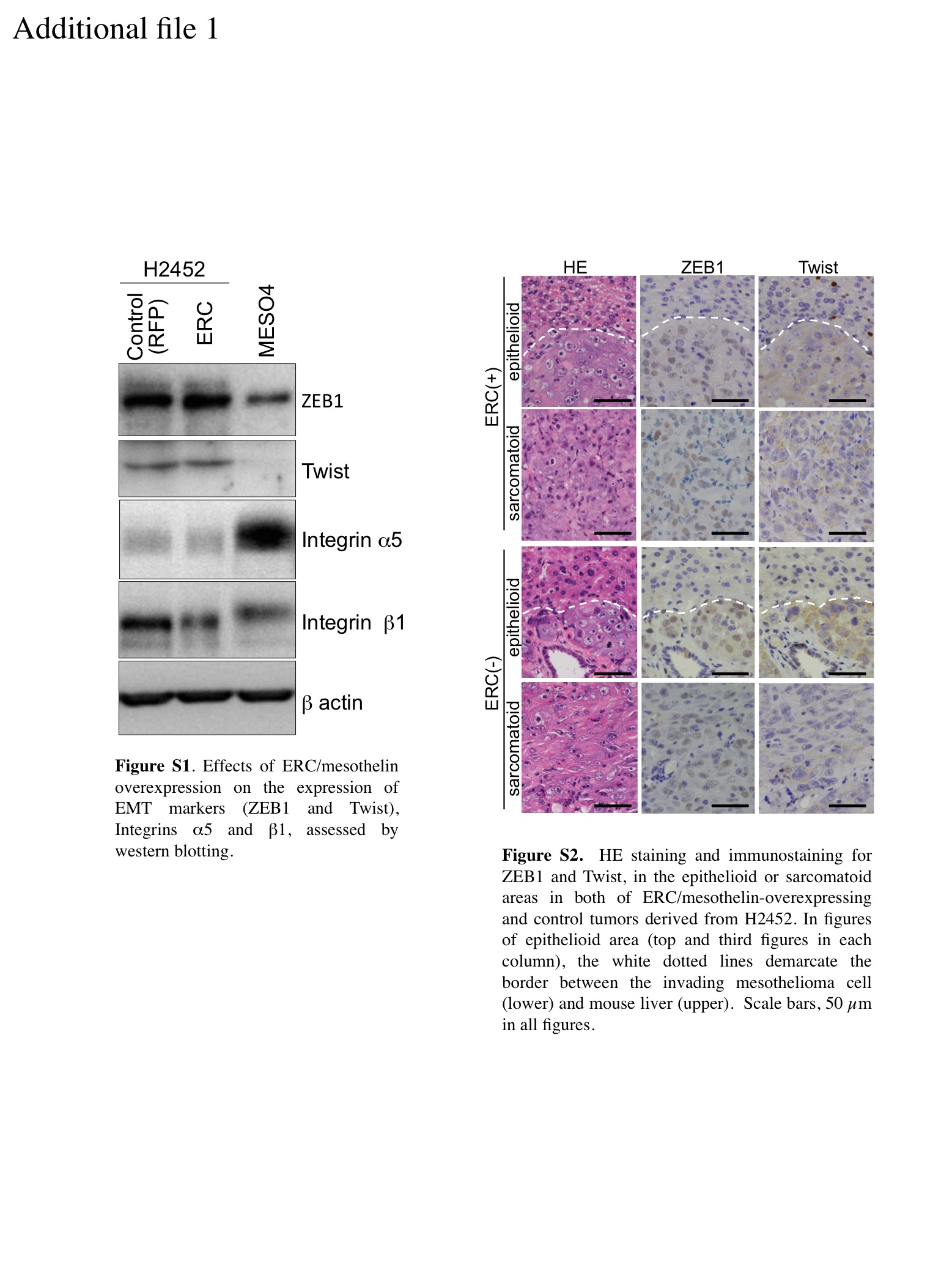

Supplement: Supplementary file 1 — Additional file 1 : Figure S1. Effects of ERC/mesothelin overexpression on the expression of EMT markers (ZEB1 and Twist), Integrins α5 and β1, assessed by western blotting. Figure S2. HE staining and immunostaining for ZEB1 and Twist, in the epithelioid or sarcomatoid areas in both of ERC/mesothelin-overexpressing and control tumors derived from H2452. In figures of epithelioid area (top and third figures in each column), the white dotted lines demarcate the border between the invading mesothelioma cell (lower) and mouse liver (upper). Scale bars, 50 μm in all figures. [file 12931_2020_1449_MOESM1_ESM.jpg]
